# The Bimalleolar Method Shows the Most Reliable Results for Measuring Tibial Torsion in Rotational MRI

**DOI:** 10.3390/jcm14134523

**Published:** 2025-06-26

**Authors:** Klemens Vertesich, Catharina Chiari, Martin Zalaudek, Karin Hebenstreit, Eleonora Schneider, Reinhard Windhager, Madeleine Willegger

**Affiliations:** 1 Department of Orthopedics and Trauma Surgery, Medical University of Vienna, Waehringer Guertel 18-20, 1090 Vienna, Austria; klemens.vertesich@meduniwien.ac.at (K.V.); karin.hebenstreit@gmx.at (K.H.); eleonora.schneider@meduniwien.ac.at (E.S.); reinhard.windhager@meduniwien.ac.at (R.W.); madeleine.willegger@meduniwien.ac.at (M.W.); 2 Department of Pediatric Orthopaedics and Foot and Ankle Surgery, Orthopedic Hospital Speising, Speisinger Straße 109, 1130 Vienna, Austria; 3 Department of Biomedical Imaging and Image-guided Therapy, Medical University of Vienna, Waehringer Guertel 18-20, 1090 Vienna, Austria

**Keywords:** tibial torsion, rotational MRI, torsional malalignment of the lower extremity, rotational osteotomy

## Abstract

**Background**: The reproducible measurement of tibial torsion (TT) is essential for the diagnosis and evaluation of rotational deformities of the tibia, particularly in the planning of tibial derotational osteotomy. While various CT-based methods for determining the distal tibial axis have been described for adult patients, rotational Magnetic Resonance Imaging (MRI) represents a radiation-free alternative, especially for assessing lower limb rotation in pediatric patients. The aim of this study was to analyze the reliability of TT measurements as well as to investigate potential differences in the application of rotational MRI within a pediatric orthopedic cohort. **Methods**: In this retrospective study, 78 lower legs from 39 patients aged 4 to 18 years who underwent rotational MRI were included. Measurements for TT were performed using the Jend method, the Waidelich method, and the bimalleolar method. Reliability assessments were conducted by three different examiners, and the results were determined using the intraclass correlation coefficient (ICC). **Results**: All three methods demonstrated excellent interobserver reliability. The highest intraobserver reliability was achieved using the bimalleolar method (ICC: 0.947). When comparing the assessment of TT, the Jend method showed the highest mean values (34°, standard deviation (SD) 11.0°) followed by the Waidelich method (29°, SD 10.2°) and the bimalleolar method (26°, SD 9.9°). Measurement methods showed a mean difference of up to 8° (*p* < 0.001). **Conclusions**: Rotational MRI is a feasible radiation-free option to assess tibial torsion in pediatric and adolescent patients. All tested methods show excellent inter- and intraobserver reliability. Notably, significant differences were found between the measurement methods, with the bimalleolar method showing lower mean values. This has to be taken into account for preoperative planning of rotational and derotational tibial and supramalleolar osteotomies.

## 1. Introduction

Variations in tibial torsion (TT) cause pathological gait patterns (i.e., toeing-in and toeing-out) in children [1,2]. Altered gait mechanics are often the origin of pain around the foot and ankle or knee. Other functional disabilities such as patella instability can also be induced by rotational malalignment [3]. During longitudinal bone growth, torsional malignment of the tibia might spontaneously correct itself to a certain degree and age, but post-traumatic deformities or excessive external TT in children with congenital clubfoot or neurological disorders are especially common indications of rotational and derotational tibial osteotomies [4,5,6]. The choice of the height of the tibial osteotomy must be critically evaluated and planned.

Proximal tibial osteotomies enable biplanar deformity correction but are generally limited to corrections of 15° or less due to challenges with fixation and the risk of neurovascular injury [7]. Peroneal nerve neurolysis may be considered, particularly for larger corrections [8]. Fixation is typically achieved using plates, which often necessitate postoperative weight-bearing restrictions [9]. Midshaft osteotomies with intramedullary nailing are primarily suited for rotational deformities, though minor coronal corrections may be feasible if the osteotomy is positioned more distally [10]. Prophylactic anterior compartment fasciotomy is commonly performed to reduce the risk of compartment syndrome [11,12]. Intramedullary fixation allows for early weight bearing and unrestricted range of motion postoperatively [11]. The supramalleolar region of the distal tibia is the most favorable site for performing rotational osteotomies of the tibia [13]. It is considered safer compared to other alternatives of proximal osteotomies with a reduced risk of neural injuries. However, complications remain notable, with reported major complication rates ranging between 5% and 10%, with residual deformities occurring in a small percentage of cases [13,14]. Therefore, optimal planning is crucial. Supramalleolar osteotomies are commonly stabilized using either plate fixation [13,15], crossed K-wires and casting [16] (particularly in smaller children), or external fixation [15]. Postoperative weight bearing and range of motion depend on the chosen method of stabilization [8,13]. Robust evidence regarding the necessity of a fibular osteotomy remains limited [17]. However, it is argued that performing a fibular osteotomy can enhance the mobility of the tibial osteotomy and facilitate more extensive corrections. This may be particularly beneficial in cases where achieving the targeted degree of correction proves challenging intraoperatively [11,13,15].

The basic assessment of the rotational profile of the lower extremities is part of a thorough pediatric orthopedic exam including a visual gait assessment together with the foot progression angle (FPA) and evaluation of the thigh–foot angle, as well as the tibial torsion by use of the tibial tubercule and the transmalleolar axis as anatomical landmarks [18,19,20,21,22,23].

If surgical correction of the tibial torsion is planned, further radiographic assessment is suggested to quantify the amount of deformity. Rotational Computed Tomography (CT) is considered the gold standard of assessment, as it shows accurate und reproducible results [24,25]. Rotational CT scans include sections through the hip, knee, and ankle joint of both lower extremities. TT is assessed by measuring the angle between the posterior margin of the tibial plateau and another distal tibial axis. In the literature, several methods were described which vary in the anatomical landmarks of the distal tibial axis. However, the most commonly used methods are described by Jend, Waidelich, and the bimalleolar method [24,26,27].

All three techniques utilized a common proximal reference axis, defined as a tangent to the dorsal margin of the tibial plateau, positioned 3–6 mm proximal to the tip of the fibula. The methods differ primarily in how the distal tibial axis is determined. The technique described by Jend et al. defines the distal axis on a plane above the medial malleolus by connecting the midpoint between the anterior and posterior margins of the incisura fibularis to the center of the tibial pilon, approximated by a fitted circle [26]. In contrast, the Waidelich et al. method uses an ellipse-based approach, placing ellipses along the incisura fibularis and the surface of the medial malleolus, with the axis drawn through the centers of these ellipses [27]. The bimalleolar method takes a more direct anatomical approach, defining the axis by connecting the centers of the medial and lateral malleoli on an axial image showing both structures clearly [24]. Compared to the Jend and Waidelich techniques, which rely on geometric approximations and specific anatomical landmarks of the incisura region, the bimalleolar method follows a more direct anatomical approach but may be more susceptible to variability due to differences in malleolar morphology or positioning. Although modern low-dose CT assessment techniques show significantly reduced radiation, this investigation shows that they still cause notable radiation exposure to pediatric patients [28,29].

Magnetic Resonance Imaging (MRI) represents a radiation-exposure-free option, which would be preferred in a pediatric and adolescent patient population. CT-based measurement methods for the assessment of femoral and tibial torsion have been proven to be reliable on rotational MRI in adults [25,30,31,32]. To date, there are limited data on the feasibility, accuracy, and reliability of rotational MRI in pediatric patients. Depending on age, their local anatomy at the proximal tibia and the supramalleolar region might be different from adult anatomy.

This study aimed to assess the feasibility of MRI-based rotational assessments of TT in pediatric patients using three common measurement techniques. Furthermore, the reliability and accuracy of TT measurements were assessed and an investigation of the most reliable method was performed.

## 2. Methods

This study was approved on 26 September 2019 by the ethics committee of the Medical University of Vienna (Nr. EK Nr: 1805/2018). Rotational MRI is routinely performed at our institution as of January 2014 according to a standardized protocol. Pediatric and adolescent patients who consulted the department’s outpatient clinic for rotational malalignment were routinely evaluated using the rotational MRI of both lower extremities. TT measurements were prospectively assessed on these patients. Exclusion criteria were ages younger than 4 and older than 18 years, those with clinical coronal malalignment of 15° or more, severe deformity of any part of the lower limb, a leg length discrepancy of 2.5 cm or more, or a history of surgery for rotational or coronal malalignment. These criteria were established to ensure a more homogeneous study population and to minimize potential measurement biases.

Thirty nine patients (78 lower legs) with a mean age of 11.8 years (SD 3.66, range 4–18) were included in this study. Thirty two patients presented with clinical suspicion of malrotation, three presented with knee pain, three presented with hip pain and one presented with recurrent patella instability and dislocation.

Investigations were performed on a Siemens Aera 1.5T MRI (Siemens Healthineers, Erlangen, Germany). A T2-weighted, TSE sequence was applied for axial pelvic, knee, and ankle image acquisition using a body coil. The patient was placed supine on the MRI table and both legs were padded and stabilized with sand bags in an extended hip and knee position and neutral ankle dorsiflexion. For image evaluation, DeepUnity Insight platform (Dedalus Healthcare GmbH, Milano, Italy) was used.

**Figure 1:**
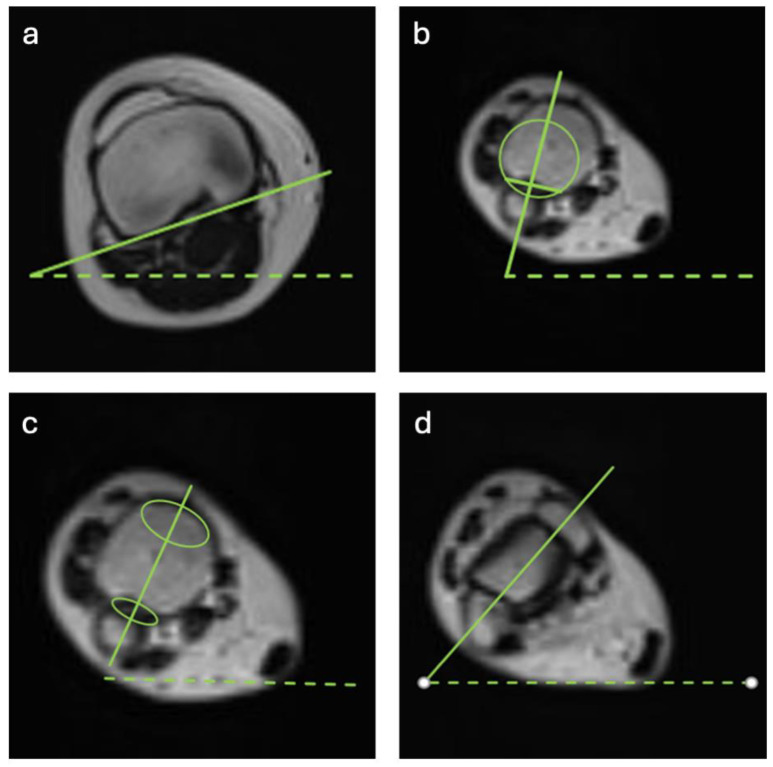
(**a**–**d**) Overview of all three different measurement techniques that differ in the distal axis. (**a**) Proximal reference where an axis is created as a tangent at the dorsal margin of the tibial plateau. (**b**) Method according to Jend et al. [26], where the axis is created by connecting the midpoint of a line between the anterior and posterior edges of the incisura fibularis and the center of the tibial pilon, estimated by a circle. (**c**) Method according to Waidelich et al. [27] with the use of an ellipse along the incisura fibularis and an ellipse on the surface of the medial malleolus with a line through the centers of these two ellipses, creating the axis. (**d**) Bimalleolar method by visualizing both malleoli and creating an axis through the centers of the medial and lateral malleoli.

TT was assessed on axial T2 MRI images by using three different techniques. All techniques use the same proximal axis, which is defined by a tangent of the dorsal margin of the tibial plateau 3–6 mm proximal from the tip of the fibula. The distal axis was measured as follows (Figure 1): First, the method according to Jend et al. was assessed on a plane above the medial malleolus [26]. The axis was created by connecting the midpoint of a line between the anterior and posterior edges of the incisura fibularis and the center of the tibial pilon, estimated by a circle [26]. Second, the method according to Waidelich et al. was employed, which uses an ellipse along the incisura fibularis and an ellipse on the surface of the medial malleolus [27]. A line through the centers of the two ellipses created the axis [27]. And third, the bimalleolar method was performed on a slice that visualizes both malleoli. Then, an axis was drawn through the centers of the medial and lateral malleoli [24].

Assessments were performed by three different observers (a pediatric orthopedic surgeon (MW), a musculoskeletal radiologist (MZ), and a trained medical student (KS)). To avoid systematic measurement errors, all measurements were performed as a training exercise on a cohort of 10 adult patients by all three observers. The measurements were conducted twice by one of the observers in two different sessions within a time interval of 2–3 weeks.

### Statistical Analysis

Intraclass correlation coefficient (ICC) was used to assess inter- and intraobserver reliability. ICC results were considered as having poor reliability for values under 0.5, moderate reliability between 0.5 and 0.75, good reliability between 0.75 and 0.9, and excellent reliability for values of 0.9 and above [33]. ICCs were visualized by Bland–Altman plots. Sample size calculation was performed with Bonnet’s approximation, using an ICC targeted as 0.8, a 95% Confidence Interval (CI), and three observers. The calculation resulted in a minimum sample size of 36 extremities. Descriptive statistics were used to assess mean values and standard deviation (SD) of measurement results. Further, the differences between the measurement methods were compared using Analysis of Variance (ANOVA) with post hoc Bonferroni correction. All statistical assessments were performed using SPSS 29.0 (IBM Co., Armonk, NY, USA).

## 3. Results

**Figure 2:**
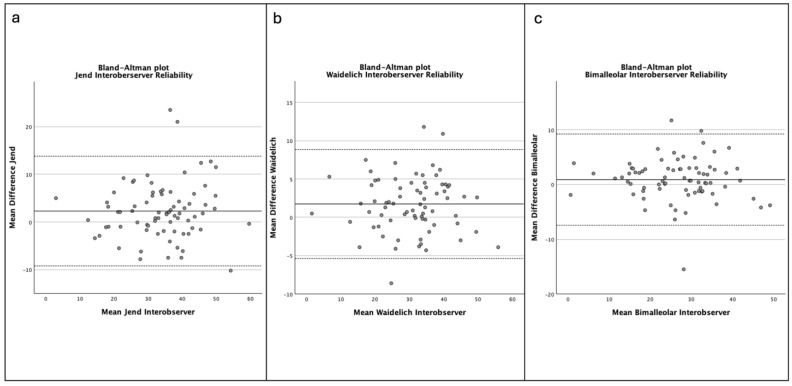
(**a**–**c**). Bland–Altman plots displaying interobserver agreement of the methods ((**a**)—Jend method, (**b**)—Waidelich method, (**c**)—bimalleolar method) for the radiographic measurement of the tibial torsion (TT). The solid line represents the mean difference in measurements between the observers. The dashed lines represent the 95% limits of agreement.

All methods showed excellent reliability with high ICC values (Table 1). The interobserver reliability of the bimalleolar method showed an ICC of 0.996 (95% CI 0.950–0.977). Equally, the method according to Waidelich et al. [27] showed an interobserver reliability of 0.996 (95% CI 0.950–0.977). The method according to Jend et al. [26] showed an interobserver reliability calculated with an ICC of 0.933 (95% CI 0.903–0.955) (Figure 2a–c).

**Figure 3:**
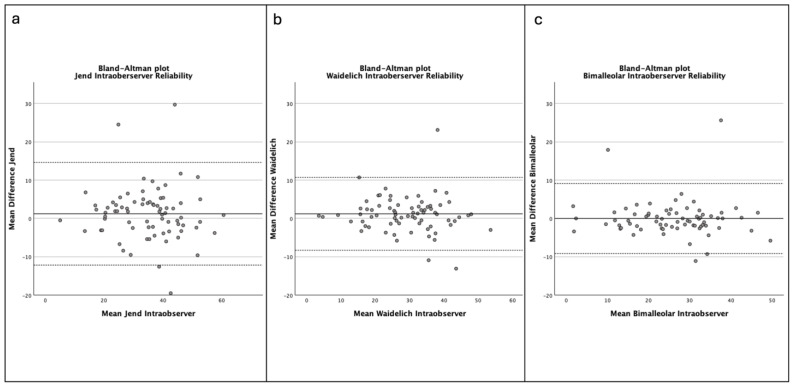
(**a**–**c**). Bland–Altman plots showing intraobserver agreement of the methods ((**a**)—Jend method, (**b**)—Waidelich method, (**c**)—bimalleolar method) for the radiographic measurement of the tibial torsion (TT). The solid line represents the mean difference in measurements between the two measurement time points. The dashed lines represent the 95% limits of agreement.

The bimalleolar method showed superior intraobserver reliability with an ICC of 0.947 (95% CI 0.916–0.966). The ICC for intraobserver reliability was 0.942 (95% CI 0.909–0.963) for the Waidelich method and 0.903 (95% CI 0.848–0.938) for the Jend method, respectively (Figure 3a–c).

**Figure 4:**
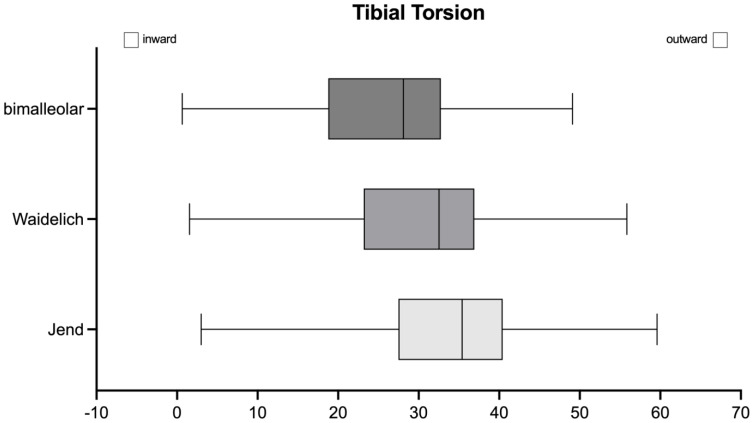
Visualization of the three different methods of the tibial torsion (TT) assessment showing differences in means between the methods (dark grey—bimalleolar method, grey—Waidelich method, light grey—Jend method) as well as an indication of the overestimation of external or outward rotation of the Jend or Waidelich method compared to the bimalleolar method.

Upon comparing the mean values of TT, the bimalleolar method showed the lowest mean with 26° (SD 9.9°), followed by the Waidelich method with a mean of 29° (SD 10.2°) and the method according to Jend et al. with a mean of 34° (SD 11.0°), leading to a maximum difference of 8° between the methods (Figure 4). The mean TT showed significant differences between the methods assessed by ANOVA (*p* < 0.001).

## 4. Discussion

Radiographic assessment of TT is becoming more popular and common among pediatric orthopedic surgeons to quantify rotational malalignment. A rotational radiographic evaluation in terms of a rotational CT or MRI should be mandatory in addition to the clinical exam not only for medical–legal reasons, but also for the accurate planning of the amount of torsional correction [34]. For the surgical planning of derotational and rotational SMO, the preoperative TT measurement has a direct impact on the extent of the planned correction [35]. Some surgeons advocate for additional fibular osteotomy when excessive correction is indicated [36]. Cross-sectional CT scans are still considered the gold standard for TT assessment [24,37]. However, several recent cadaveric and clinical studies have shown high correlation between CT- and MRI-based measurements [25,38,39]. Especially in pediatric and adolescent patients, the reduction in radiation exposure is of major concern, therefore implementing an MRI protocol in clinical practice for rotational limb analysis is favorable.

The current study proved the reproducibility and showed a reliable measurement of TT in pediatric patients using rotational MRI. It showed the most commonly used methods and showed excellent inter- and intraobserver reliabilities when assessing TT.

Inter- and intraobserver reliability could be considered excellent with an ICC above 0.9. Liodakis et al. investigated the same three different methods for TT based on CT scans in adults also achieving excellent ICCs for intra- and interobserver classification [24]. Further, our results compared to Liodakis et al. favor the bimalleolar method over the Jend and Waidelich methods in terms of reliability with superior ICCs for both intra- and interobserver reliability. The bimalleolar method is straightforward and efficient for examiners, as it enables a direct identification of the distal axial plane by accurately estimating the positions of the medial and lateral malleoli—a process that is readily achievable in clinical practice. Methods according to Jend and Waidelich rely on more graphical figures for the estimation of the radiological landmarks and, in that regard, may be more difficult in assessment and more time-consuming [24,25,26,27]. Despite the fact that the malleoli are still cartilaginous in children and the identification of structures is more challenging than in adult patients, the bimalleolar method shows the most reliable results.

Basaran et al. investigated TT on MRI in 17 pediatric patients. The authors describe a lack of feasibility in their pediatric population when using the methods by Jend et al. and Waidelich et al. despite prior use of MRI for adults in other studies [40]. Therefore, these two common methods were not assessed in their study. The authors used, as a distal reference, an axis through the posterior intermalleolar region as one method and the anterior border of the talus as the second method in addition to the bimalleolar method. Their results favored the anterior border of the talus as the distal reference compared to the other methods. Despite the use of MRI with the same field strength, image acquisition in the current study was performed using T2-weighted TSE sequences. This sequence enables sufficient image acquisition and displays anatomical landmarks of the distal tibia and fibula, allowing for reliable assessment using not only the bimalleolar method, but also the Jend and Waidelich methods. Conversely, the bimalleolar method still showed the most accurate results in terms of inter- and intraobserver reliability.

Excessive tibial torsion correlates with clinical problems presumably around the knee joint such as anterior knee pain, gait disturbances, or patellar instability [3,41,42]. Barton et al. recently showed that an abnormal TT of only 30° external torsion can already cause patellar maltracking or instability where potential correction osteotomy should be considered [6]. Further, studies have shown that excessive external rotation also affects statics of the foot and may lead to early development of flatfoot deformity [4]. Still of debate is the range of the normal tibial torsion, especially for children. Jacquernier et al. described the rotational profile of healthy children with the clinical method of Staheli et al. [18]. The study showed a mean increase in TT of approximately 2° in 3- to 10-year-old patients. However, studies with CT- or MRI-based assessments did not implement these or obtain similar findings to describe an age-related normal range of TT. Ultimately, a range from 5 to 40° of external TT is considered normal. Importantly, all the described methods over- or underestimate TT depending on the level of the distal measurement axis, which may be attributed to the use of different anatomical landmarks [17,42]. The results from our study verify these findings through the rotational MRI of pediatric patients. Significant differences could be found in the mean values of TT with a difference of more than 8° between the Jend method and the bimalleolar method. This fact has to be taken into account when assessing TT and especially when considering surgical correction. Concordant to other studies, we recommend using one method throughout clinical practice and recording the used method [17,43]. The bimalleolar method is easy to employ and therefore also quicker to use for assessments. The Jend method might be at risk of overestimating the TT. Accurate preoperative assessment of rotational deformities is key for effective surgical planning. Intraoperatively, achieving the intended degree of rotational correction depends largely on the surgical technique employed [13]. The optimal site for osteotomy remains a topic of ongoing debate. However, given the comparatively lower risk of complications, particularly neural injury, associated with supramalleolar osteotomies [14], this approach is preferred by the authors. To guide rotational correction, K-wires are inserted proximal and distal to the planned osteotomy site to define the desired rotational axis prior to performing a horizontal osteotomy [14]. Intraoperative assessment of the achieved correction is typically conducted using angular plates or other mechanical guides. Recently, the use of smartphone-based applications has been proposed to enhance the precision of rotational corrections. While further research is needed to validate these technologies, preliminary findings suggest that such tools may offer promising improvements in surgical accuracy [44,45].

Several limitations of this study have to be mentioned. Although imaging and data were prospectively collected and evaluated, the retrospective design of the study introduces a potential risk of selection bias. This may have influenced the composition of the study population and, consequently, the interpretation of the results. All included patients presented to our outpatient clinic with pain and issues that could potentially be affected by malrotation. Abnormal rotation was therefore present in a limited number of patients. In addition, the exclusion criteria served to homogenize the patient population by omitting cases with severe deformities. This aspect of the study design aimed to reduce potential measurement bias, as severe deformities may compromise MRI reconstruction quality and hinder the clear visualization of standardized anatomical landmarks. Although efforts were taken to optimize MRI, pediatric bones still present with cartilaginous structures that might be challenging for defining anatomical landmarks relevant for measurements. Nevertheless, even after performing measurements in one training session, a medical student was able to perform the measurements as accurately as a pediatric orthopedic surgeon and a musculoskeletal radiologist. Furthermore, the acquisition time for rotational MRI is longer compared to CT. Although imaging protocols at our institution have been optimized, by reducing acquisition time to approximately 20 min, sedation may still be required, particularly in younger or less cooperative pediatric patients, to ensure accurate and reproducible imaging. This should be acknowledged as a potential limitation of the technique. The higher cost associated with MRI may represent an additional limitation. While MRI accessibility is gradually improving, it remains limited in many regions and healthcare systems worldwide. Despite these challenges, this study demonstrates that rotational measurements obtained by MRI are both safe and reliable, offering a valuable alternative to CT that avoids ionizing radiation. In settings where MRI is not readily available due to financial, logistical, or infrastructural constraints, CT continues to serve as the standard imaging modality for rotational assessment.

## 5. Conclusions

All three radiographic assessment methods proved to be particularly reliable, with excellent inter- and intraobserver reliability for measuring tibial torsion (TT) in rotational MRI in pediatric patients. Nevertheless, significant differences in the mean values of TT were detected with the bimalleolar method showing lower values for TT. This study quantifies measurement differences which should be taken into account when assessing TT on rotational MRI for preoperative planning.

## Figures and Tables

**Table 1:** Overview of interclass correlation for inter- and intraobserver reliability.

	Interobserver Reliability	Intraobserver Reliability
	ICC (95% CI)	ICC (95% CI)
Jend	0.933 (0.903–0.955)	0.903 (0.848–0.938)
Waidelich	0.966 (0.950–0.977)	0.942 (0.909–0.963
bimalleolar	0.966 (0.950–0.977)	0.947 (0.916–0.966)

## Data Availability

The data supporting the findings of this study are presented within the manuscript. Additional details are available from the corresponding author upon reasonable request, subject to institutional and ethical guidelines.

## Abbreviations

The following abbreviations are used in this manuscript:

ANOVA	Analysis of Variance
CI	Confidence Interval
CT	Computed Tomography
FPA	Foot Progression Angle
ICC	Intraclass Correlation Coefficient
MRI	Magnetic Resonance Imaging
SD	Standard Deviation
TT	Tibial Torsion
